# Molecular Analysis of Non-Specific Protection against Murine Malaria Induced by BCG Vaccination

**DOI:** 10.1371/journal.pone.0066115

**Published:** 2013-07-04

**Authors:** Marcela Parra, Xia Liu, Steven C. Derrick, Amy Yang, Jinhua Tian, Kristopher Kolibab, Sanjai Kumar, Sheldon L. Morris

**Affiliations:** Center for Biologics Evaluation and Review, USFDA, Bethesda, Maryland, United States of America; Agency for Science, Technology and Research - Singapore Immunology Network, Singapore

## Abstract

Although the effectiveness of BCG vaccination in preventing adult pulmonary tuberculosis (TB) has been highly variable, epidemiologic studies have suggested that BCG provides other general health benefits to vaccinees including reducing the impact of asthma, leprosy, and possibly malaria. To further evaluate whether BCG immunization protects against malarial parasitemia and to define molecular correlates of this non-specific immunity, mice were vaccinated with BCG and then challenged 2 months later with asexual blood stage *Plasmodium yoelii* 17XNL (PyNL) parasites. Following challenge with PyNL, significant decreases in parasitemia were observed in BCG vaccinated mice relative to naïve controls. To identify immune molecules that may be associated with the BCG-induced protection, gene expression was evaluated by RT-PCR in i) naïve controls, ii) BCG-vaccinated mice, iii) PyNL infected mice and iv) BCG vaccinated/PyNL infected mice at 0, 1, 5, and 9 days after the *P. yoelii* infection. The expression results showed that i) BCG immunization induces the expression of at least 18 genes including the anti-microbial molecules lactoferrin, eosinophil peroxidase, eosinophil major basic protein and the cathelicidin-related antimicrobial peptide (CRAMP); ii) an active PyNL infection suppresses the expression of important immune response molecules; and iii) the extent of PyNL-induced suppression of specific genes is reduced in BCG-vaccinated/PyNL infected mice. To validate the gene expression data, we demonstrated that pre-treatment of malaria parasites with lactoferrin or the cathelicidin LL-37 peptide decreases the level of PyNL parasitemias in mice. Overall, our study suggests that BCG vaccination induces the expression of non-specific immune molecules including antimicrobial peptides which may provide an overall benefit to vaccinees by limiting infections of unrelated pathogens such as *Plasmodium* parasites.

## Introduction

*Mycobacterium bovis* BCG has been used globally as a vaccine against tuberculosis (TB) for more than eight decades. Although the effectiveness of BCG vaccination in preventing pulmonary TB is uncertain because efficacy estimates from controlled clinical trials have varied between 0–80%, BCG has consistently been shown to protect against severe disseminated forms of TB in infants [1], [2]. Interestingly, despite its questionable effectiveness in preventing TB, early observations suggested that immunization with BCG conferred an overall beneficial impact on childhood survival [3]. In more recent years, several studies have supported these initial findings by showing that BCG vaccination imparts wide ranging beneficial health-related effects that are not directly related to its anti-tuberculosis activity. For instance, epidemiologic data has shown that BCG vaccination was associated with a 45% decrease in infant mortality in Guinea-Bissau and Benin [4], [5]. This non-specific effect of BCG immunization transcended its impact on reducing disseminated childhood TB. Additionally, case control studies in Brazil demonstrated that BCG immunization reduced the risk of pneumonia-related deaths by 50% [6]. A meta-analysis of 23 studies showed that exposure to BCG in early life was also associated with a protective effect against the development of asthma [7]. For several decades, intravesical BCG therapy has been the treatment of choice for several forms of bladder cancer because of the potent anti-tumor activity of BCG [8].

The antimicrobial activity against non-TB pathogens seen after BCG immunization may contribute to the non-specific beneficial public health impact of BCG vaccine. Experiments in multiple animal models have shown that BCG immunization confers partial protection against unrelated pathogens including *Babesia microti*, *Plasmodium berghei*, *Toxoplasma gondhi*, and *Trypanosoma cruzi* [9], [10], [11], [12]. In humans, clinical trial data has indicated that BCG vaccination protects against paucibacillary as well as multibacillary leprosy; the magnitude of the anti-leprosy protection has been estimated to be 41% [13]. The increased risk of *M. avium* infections seen in the Czech Republic when BCG immunization of newborns was discontinued suggested that BCG may also provide protection against disease caused by *M. avium* complex bacilli [14]. Furthermore, an observational study in Guinea Bissau concluded that the presence of a BCG scar in children significantly reduced the risk of death from malaria [5].

The immune mechanisms associated with the non-specific beneficial effect of BCG immunization are unknown. It has been shown that BCG immunization creates a Th1-like immune environment where cytokines such as IFN-γ and IL-12 and related chemokines including Cxcl9 and Cxcl10 are over-expressed [15], [16]. This strong induction of Th1-type immune responses after BCG immunization may explain its impact on asthma; when Th2-type immune responses characteristic in patients with asthma are decreased, the development of atopic disorders are reduced. Importantly, BCG infection has also been shown to up-regulate the expression of antimicrobial molecules such as the cathelicidin-like peptides [17]. These immune molecules play an important role in innate host defenses via the direct killing of a wide variety of microbes and in adaptive immune responses through immune regulation [18], [19]. Often the synergism between cathelicidins and other defensin-like compounds can greatly amplify the host antimicrobial response. The capacity of BCG to stimulate innate immune mechanisms including the expression of broad spectrum antimicrobial molecules may contribute to its ability to limit infections by unrelated pathogens.

In this study, we assessed the impact BCG immunization on the progression of mouse malaria infections and evaluated the molecular factors that may contribute toward BCG-induced immunity against malaria. Similar to tuberculosis, malaria remains a significant global public health challenge with the total burden of disease estimated to be approximately 250 million clinical cases and about one million deaths, mainly in young sub-Saharan African children [20]. Clearly, because many children who are vaccinated with BCG reside in areas with high rates of malaria, it is important to investigate whether BCG induces non-specific protection against malaria. To characterize the impact that BCG immunization has on the course of malaria infections, we evaluated whether BCG vaccination protected against PyNL infections of mice. We showed that immunization with BCG partially protects against the malaria infection. In addition, we demonstrated that immunization with BCG induces the expression (even in malaria-infected mice) of at least 18 host genes including 4 genes encoding antimicrobial peptides. Finally, we showed *in vivo* that treatment with two of these antimicrobial peptides, lactoferrin and a cathelicidin-type peptide, significantly reduced the levels of parasitemia in PyNL-infected mice.

## Materials and Methods

### Murine Vaccination and *P. yoelii17XNL* Challenge Experiments

Pathogen-free C57BL/6 mice were obtained from the Jackson Laboratories (Bar Harbor, ME). For the malaria infection studies, five to ten mice per group were evaluated. First, the mice were vaccinated once subcutaneously with BCG Pasteur (10^6^ bacteria in 0.1 ml of phosphate-buffered saline). At 2 months after the BCG vaccination, the mice were challenged intraperitoneally (IP) with 10^6^ PyNL blood stage parasites. To prepare the malaria parasites, frozen stocks of PyNL-infected erythrocytes were thawed and used to infect IP three donor C57BL/6 female mice. The percent parasitemias were monitored every three days using blood smears. When 8 to 12% parasitemias were detected, blood was collected by cardiac puncture, diluted in PBS and used to infect experimental animals with 1×10^6^ PyNL parasites in 200 µl of PBS. The percent parasitemia (parasitized RBCs/total RBCs × 100) after infection was determined by examining Giesma-stained thin blood smears.

The role of CD4 and CD8 T cell subsets in the BCG vaccine induced protective responses was assessed using cell depletion procedures. For these studies, BCG vaccinated mice were treated with 0.5 mg of a αCD4 antibody (clone GK1.5) or a αCD8 antibody (clone 2.43) at 4 and 2 days before the PyNL infection. Subsequent flow cytometry analysis showed that >99% of CD4 and CD8 T cells had been depleted by the antibody treatment.

To investigate the potential impact of the over-expressed antimicrobial peptides on the *P*. *yoelii* erythrocytic infection, 10^6^ PyNL blood stage parasites were incubated for 2 hours with 50 µg/ml of lactoferrin or 1 hour with 50 µg/ml of the cathelicidin LL-37 peptide and then injected IP into naïve mice. One day later, 50 µg of lactoferrin or LL-37 was injected IP into the infected mice. The percent parasitemia was again determined by examining Giesma-stained thin blood smears.

### Analysis of Gene Expression in Spleen Cells by RT-PCR

For the *in vivo* gene expression analysis, at 2 months post BCG vaccination and at Day 1, Day 5 and Day 9 following PyNL infections, the spleens were removed from mice in each of the experimental groups (naïve, BCG, PyNL and BCG plus PyNL infected mice) and the recovered cells were stored in RNA later (Qiagen, Valencia, CA). For the PyNL “in vitro” recall response studies, spleen cells from naïve and BCG vaccinated mice were plated at a concentration of 2.5×10^6^ cells/well and incubated with 10^6^ PyNL freshly isolated parasites. After an overnight incubation, the cells were harvested and stored in RNA later.

The RNeasy mini kit (Qiagen) was used to isolate the total RNA from the cell suspensions. Equal amounts of RNA from these samples (typically about 1 µg) were reverse transcribed to cDNA using Ambion – RETROscript kit (Ambion, Austin, TX). The GAPDH control was used to determine the quality of the cDNA conversion of each sample by PCR. The cytokine/chemokine/innate transcriptional responses were determined using the cDNA as the template for RT^2^ profiler PCR array system (SABiosciences, Frederick, MD) and an Applied Biosystems ViiA 7 real time PCR detection system (Applied Biosystems, Foster City, CA). The mRNA expression levels for each gene were then normalized according to the manufacturer’s instructions (ViiA 7 real time PCR array system user manual). The relative gene expression values in cell cultures were determined by dividing the gene expression levels in experimental samples by the expression values in naïve controls [15]. This value represents the mean increase or decrease of mRNA expression compared to naïve controls [17].

### Statistical Analysis

The Graph Pad Prism 5 program was used to analyze the data for these experiments (Graph Pad Software, San Diego, CA). The BCG vaccination protection data, the RT-PCR results, and the lactoferrin and LL-37 treatment data were evaluated using the unpaired t test analysis of the Graph Pad Prism software.

## Results

### BCG Vaccination Provides Partial Protection against a *P. yoelii* 17XNL Infection

**Figure 1:**
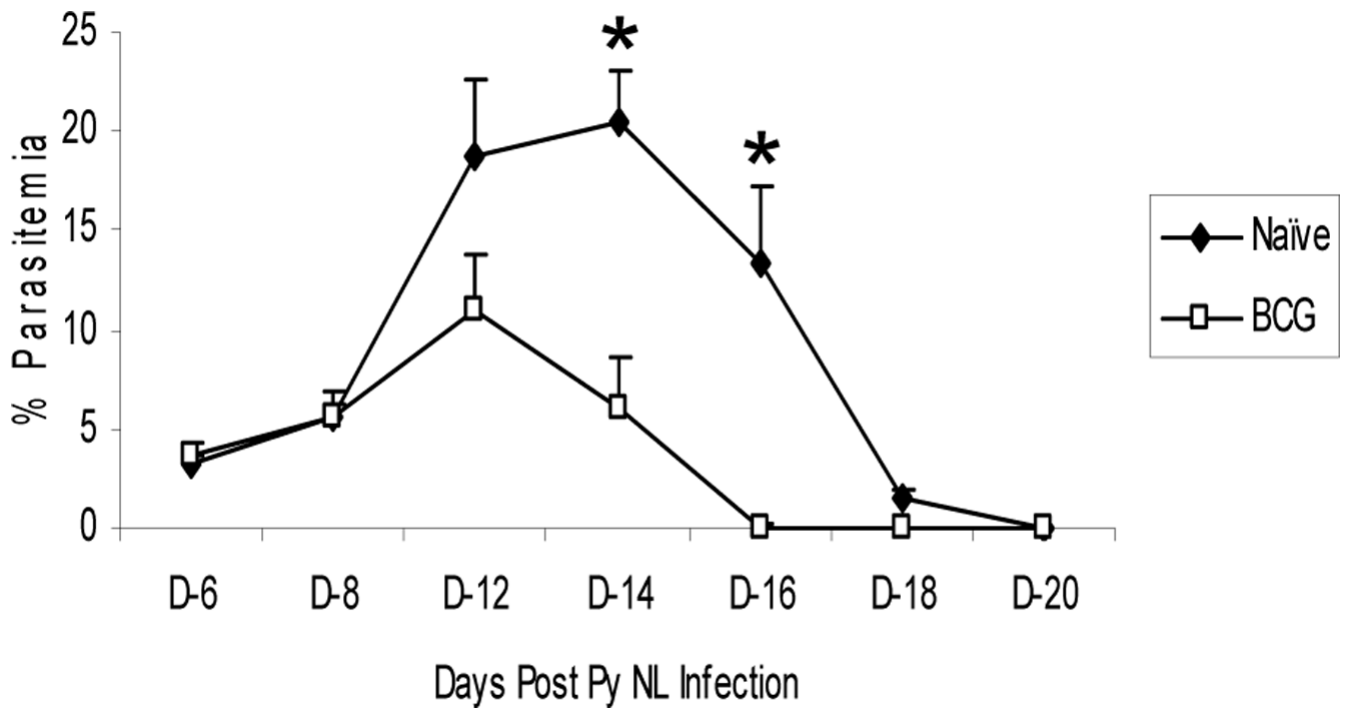
BCG vaccination confers partial protection against *P. yoelii* 17XNL infections in mice. Two months after immunization with BCG, vaccinated (closed squares) and naïve mice (closed circles) were challenged with an erythrocytic stage *P. yoelii* infection. The post-infection parasitemia levels were determined by blood smear microscopy. These data are representative results from 3 experiments. In each study, 10 mice were used for each experimental group. * Significant difference in parasitemia levels (p<0.05) as determined by unpaired t test analysis.

To evaluate whether BCG vaccination induces protection against murine PyNL infection, mice were immunized with 10^6^ CFU of BCG Pasteur and then infected with 10^6^ erythrocytic PyNL parasites at 2 months after the vaccination. Earlier studies had shown that maximal BCG-induced protective responses in mice are detected at 2 months after immunization (S. Derrick, unpublished data). A typical parasitemia curve for the non-lethal PyNL strain is shown in Figure 1. After an erythrocytic infection with the PyNL strain, the peak of parasitemia is generally observed at 12–14 days post-infection and the parasites are generally cleared at about 3–4 weeks after the infection. As shown in Figure 1, significant protection against the malaria infection was observed at 2 months post-BCG immunization (p<0.05). During this experiment, decreases in parasitemia were detected in BCG vaccinated mice at days 14 (20.5%- naïve vs. 6.0% - BCG, 71% reduction) and 16 (13.4% - naïve vs. 0.5% - BCG, 96% reduction) after the PyNL challenge. Interestingly, significant BCG-induced protection after malarial challenge was not seen when the mice were infected with PyNL parasites at 2 weeks following BCG vaccination (M. Parra, data not shown).

**Figure 2:**
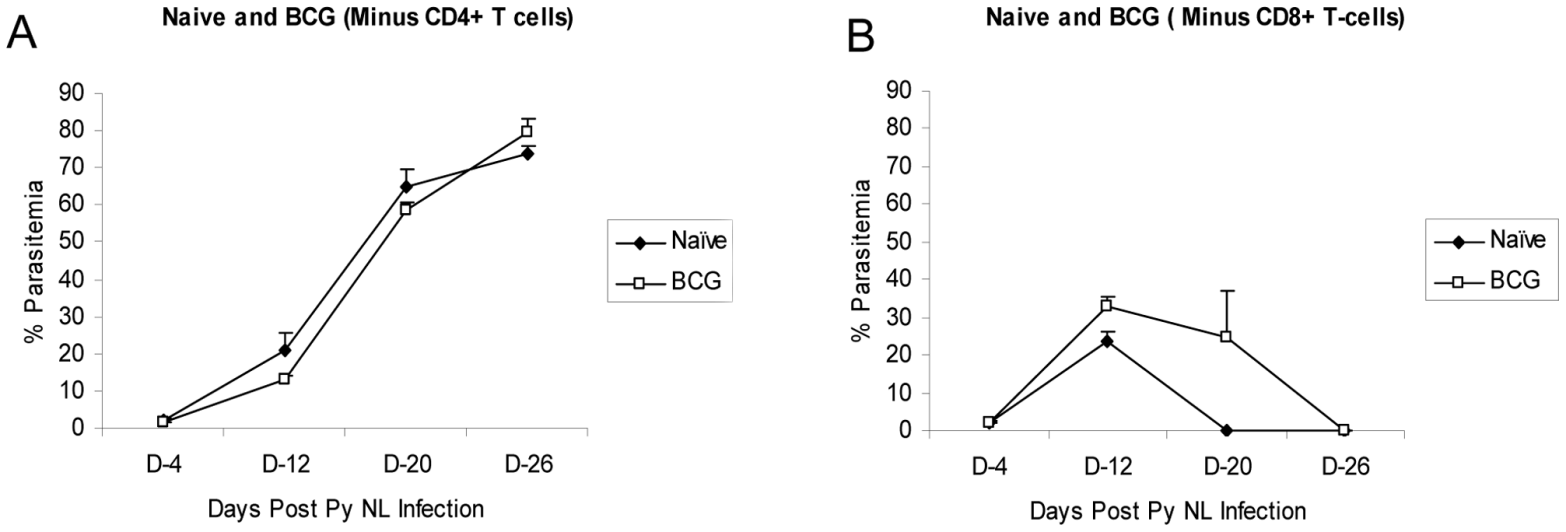
CD4 and CD8 T cells are required for the BCG vaccine induced protection against *P. yoelii* 17XNL infection. BCG vaccinated (closed squares) and naïve mice (closed circles) were injected with antibodies to deplete CD4 (A) or CD8 (B) cells and then were infected with erythrocytic stage *P. yoelii* parasites. The post-infection parasitemia levels were determined by blood smear microscopy. Ten mice were used for each experimental group. *Significant differences in parasitemia levels (p<0.05) as determined by unpaired t test analysis.

Since cellular responses are usually a critical component of the anti-malarial protective responses, we evaluated whether CD4 and CD8 T cells were responsible for the BCG-induced protection. For these studies, mice were vaccinated with BCG and then depleted of CD4 or CD8 T cells by injection of cell specific antibodies prior to the PyNL challenge. The results shown in Figure 2 indicate that both CD4 and CD8 T cells contribute to the clearance of *P. yoelii* infections in C57BL/6 mice. In the absence of CD4 T cells, both naïve and BCG-vaccinated mice fail to control and clear the PyNL infection; extremely high levels of parasitemia (>75%) were seen in CD4-depleted naïve and BCG-vaccinated mice at 26 days post-infection (Figure 2A). Since the naïve mice failed to control the PyNL infections, the impact of the CD4 depletion on BCG-induced protection is difficult to interpret. In contrast, the increases in parasitemia levels were less dramatic in mice depleted of CD8 T cells; clearance of the infection was observed at 4 weeks in CD8 depleted naïve controls and BCG-vaccinated mice (Figure 2B). However, depletion of CD8 T cells did eliminate the BCG-induced protection against the malaria challenge of C57BL/6 mice.

### Gene Expression in Malaria-infected and BCG-vaccinated Mice

To investigate the mechanisms of BCG-induced protection, gene expression experiments were initiated using the SuperArray technology and RT-PCR protocols. In these studies, the *in vivo* expression of more than 300 immune-response related genes was assessed in splenocytes from malaria infected and control mice. Since malaria infections are known to be immunosuppressive, it was not unexpected that decreased expression of 10 genes was detected at days 5 and 9 following the malaria challenge in *P. yoelii* infected mice relative to non-infected naïve controls (Table 1). Down-regulation by 2–4 fold of genes encoding immune mediators such as IFN-γ, IL-1B, IL-1m, lactoferrin (*Ltf*) and nitrous oxide synthase 2 (*Nos2*) was consistently observed at 5 and 9 days post-infection. Interestingly, as the infection progressed, up-regulation of specific genes was also detected. In particular, the significant over-expression of the *Csf1*, *Lefty1*, *Ccrl2*, *Gdf3*, *Il-10* and *Ccl8* genes (1.8–5.8 fold increases) was seen at day 9 post-infection.

**Table 1:** Relative gene expression in mice infected with *P. yoelii* and naïve controls at days 1, 5 and 9 after the malaria infection.

	Day 1			Day 5			Day 9		
	Naive	NaïvePy	Fold^a^	Naive	Naïve Py	Fold	Naive	NaïvePy	Fold
gdf15	38.2±9.9^b^	7.8±0.2	0.20	9.2±2.0	3.6±0.9	0.39	11.4±3.3	2.7±0.1	0.24
csf1	– c	–	–	8.7±0.5	4.5±0.5	0.51	6.6±0.1	33.4±4.4	5.10
lefty1	–	–	–	14.2±2.1	6.5±0.5	0.46	12.4±2.0	22.5±1.7	1.81
ifng	–	–	–	38.0±1.2	24.3±1.5	0.64	32.1±1.0	11.6±0.9	0.36
fn1	–	–	–	19.0±3.6	5.5±1.4	0.29	17.2±2.5	3.1±0.5	0.18
il1b	–	–	–	115±25	28.4±4.4	0.25	135±47.5	11.5±2.8	0.09
nos2	–	–	–	913±100	256±17	0.28	820±90	278±15	0.34
ltf	–	–	–	133±11	49.3±24.2	0.37	117±6.2	79.7±6.6	0.68
prg2	–	–	–	39.3±6.4	11.6±2.2	0.30	–	–	–
camp	–	–	–	147±11	51.9±24.4	0.35	–	–	–
il1m	–	–	–	–	–	–	4.2±1.2	0.9±0.2	0.21
ccrl2	–	–	–	–	–	–	25.8±3.6	44.4±3.4	1.8
gdf3	–	–	–	–	–	–	2.3±1.1	13.3±1.5	5.8
il10	–	–	–	–	–	–	1.9±0.3	4.2±0.1	2.2
ccl8	–	–	–	–	–	–	1.8±0.1	3.3±0.2	1.9

a Gene expression in mice infected with *P. yoelii* relative to naïve controls.

b Mean±the standard error of the mean (SEM) for 4–5 mice. For these immune mediators, significant differences were detected by unpaired t test analysis (p<0.05) in the levels of gene expression for PyNL infected and naïve mice.

c (–) Significant differences in gene expression were not detected between malaria infected mice and controls.

The impact of BCG vaccination on gene expression in non-infected and *P. yoelli* infected mice was evaluated by preparing cDNA from RNA extracted from splenocytes at days 0 (BCG vaccination control), 1, 5, and 9 days after the malaria challenge (Table 2). The expression of at least 15 genes was increased by BCG vaccination including three IL-1 related genes and several genes encoding chemokines. Importantly, the expression of four genes encoding antimicrobial peptides (*Camp*, *Ltf*, *Pgr2*, and *Epx,*) were increased 2.4 to 6.1 fold in BCG vaccinated mice at day 0. The *Ltf* gene encodes the iron-binding lactoferrin protein, the *Camp* gene encodes the multi-faceted cathelicidin-like CRAMP peptide while the two other genes encode the eosinophil proteins – major basic protein (*Pgr2*) and eosinophil peroxidase (*Epx*). The relative effect of BCG vaccination on the expression of these antimicrobial compounds continued as the active malaria infection progressed. By day 9 post-infection when the mean parasitemia levels were often about 10%, the over-expression of *Camp*, *Ltf*, *Pgr2*, and *Epx* genes remained significantly elevated compared to the PyNL infected non-vaccinated controls. Although the levels of expression for these molecules had dropped after the malaria infection 2–5 fold relative to the initial BCG-vaccinated controls, these genes were still up-regulated 1.6–3.2 fold in BCG immunized mice compared to the non-vaccinated animals.

**Table 2:** Relative gene expression in BCG vaccinated, *P. yoelii* infected mice compared to non–immunized controls.

	Day 0			Day 1			Day 5			Day 9		
Gene	naive	BCG	fold^a^	Py	PyBCG	fold^b^	Py	PyBCG	fold	Py	PyBCG	fold
Camp	241±74^c^	1225±175	5.1	150±28	1455±492	9.7	44.0±19	265±30	6.0	99.0±8.8	243±34	2.5
Ltf	208±59	1214±253	5.8	135±28	1847±343	13.7	23.5±4.2	228±23	9.7	80.0±6.5	251±38	3.2
Pgr2	58±10	137±27	2.4	43.2±10	225±75	5.2	10.9±1.7	30.4±2.5	2.8	30.8±0.3	47.8±3.6	1.6
Epx	2.2±0.6	13.6±3.1	6.1	3.2±0.9	20.8±2.5	6.1	0.9±0.3	2.6±0.2	2.8	2.9±0.4	6.9±1.4	2.4
Illb	131±18	307±45	2.3	97.1±5.7	330±74	3.4	29.5±3.3	82.0±19.3	2.8	–	–	–
Il1f9	3.9±0.5	13.9±1.5	3.6	2.7±0.3	8.8±2.4	3.3	–	–	–	–	–	–
Il1m	3.8±0.4	8.5±0.8	2.2	2.2±0.3	7.4±0.8	3.4	–	–	–	–	–	
												
Ccl2	7.9±1.1	15.8±2.7	2.0	9.3±1.3	26.2±6.1	2.8	–	–	–	–	–	–
Ccrl2	32.5±4.2	54.9±5.1	1.7	33.5±5.8	67.8±5.4	2.0	–	–	–	–	–	–
Cxcl8	–d	–	–	0.9±0.2	5.2±1.6	5.8	2.3±0.4	8.2±2.0	3.6	3.2±0.2	9.9±2.4	3.1
Cxcl9	1.9±0.3	3.0±0.5	1.6	–	–	–	–	–	–	–	–	–
Cxcl10	24.8±1.5	41.4±3.9	1.7	–	–	–	–	–	–	–	–	–
Csf1	7.9±0.6	14.4±1.1	1.8	–	–	–	–	–	–	–	–	–
Ifng	39.8±2.8	57.6±6.8	1.5	60.3±3.5	120±19	2.0	–	–	–	–	–	–
Il27	3.0±0.5	7.8±0.8	2.6	2.0±0.3	8.4±1.8	4.2	–	–	–	–	–	–
Gdf3	2.0±0.4	4.0±0.5	2.0	–	–	–	–	–	–	–	–	–
Fn1	16.2±1.6	34.3±2.7	2.1	–	–	–	–	–	–	–	–	–
Gdf15	–	–	–	7.8±0.2	21.6±4.2	4.2	–	–	–	–	–	–
Lefty1	–	–	–	13.1±1.9	23.5±2.3	1.8	–	–	–	–	–	–

a Gene expression in BCG vaccinated mice compared to naïve controls.

b Gene expression in BCG vaccinated *P. yoelii* infected mice compared to non–immunized, malaria infected controls at days 1, 5, and 9 post–infection.

c Mean±SEM for 4–5 mice. For these immune mediators, significant differences in gene expression were detected by unpaired t test analysis (p<0.05) between BCG vaccinated and naïve mice or BCG vaccinated, PyNL infected mice and non–infected PyNL infected controls.

d (–) Significant differences were not detected between vaccinated mice and controls.

To further evaluate the impact of BCG immunization on the expression of genes encoding antimicrobial molecules, splenocytes were recovered from naïve and BCG immunized mice and then were cultured overnight with and without PyNL parasitized erythrocytes. As shown in Table 3, spleen cells from BCG-vaccinated animals expressed 3–4 fold higher levels of RNA from the *Camp*, *Ltf, Pgr2*, and *Epx* genes than naïve mice. After incubation with malaria parasitized erythrocytes, increased gene expression of 3.3–7.6 fold was detected in BCG vaccinated mice for the same genes (BCG-Py vs. naïve-Py). Interestingly, while significant reductions in *Camp*, *Ltf*, and *Pgr2* gene expression (p<0.01) was seen in naïve cells incubated with *Plasmodium* infected erythrocytes relative to non-treated naïve cells (Naïve vs. Naïve-Py), the levels of gene expression were not significantly decreased for any of the tested genes in splenocytes from BCG vaccinated mice incubated with parasitized red cells.

**Table 3:** Gene expression from in vitro cultures of splenocytes recovered from naïve and BCG vaccinated mice and then stimulated with and without *P. yoelii* parasitized erythrocytes.

	Naive	BCG	fold^a^	Naïve–Py^b^	BCG–Py^b^	Fold^c^
camp	35.3±3.1^d^	129.2±18.8	3.7	20.9±2.3	158.5±25.5	7.6
ltf	52.3±4.9	212.0±34.6	4.1	30.0±4.4	210.9±27.2	7.0
pgr2	11.8±1.1	40.7±6.6	3.4	7.3±1.3	50.7±9.6	6.9
epx	2.3±0.7	7.6±1.6	3.4	2.3±0.6	7.6±1.7	3.3

a Gene expression in BCG vaccinated mice compared to naïve controls.

b Stimulated with *P. yoelii* parasitized erythrocytes.

c Gene expression in BCG vaccinated *P. yoelii* infected mice compared to non–immunized,

malaria infected controls.

d Significant differences in the levels of gene expression (Mean±SEM) were detected by unpaired t test analysis (p<0.05).

### Treatment with Lactoferrin or a Cathelicidin-type Peptide Reduces the Levels of *P. yoelii* Parasitemias

**Figure 3:**
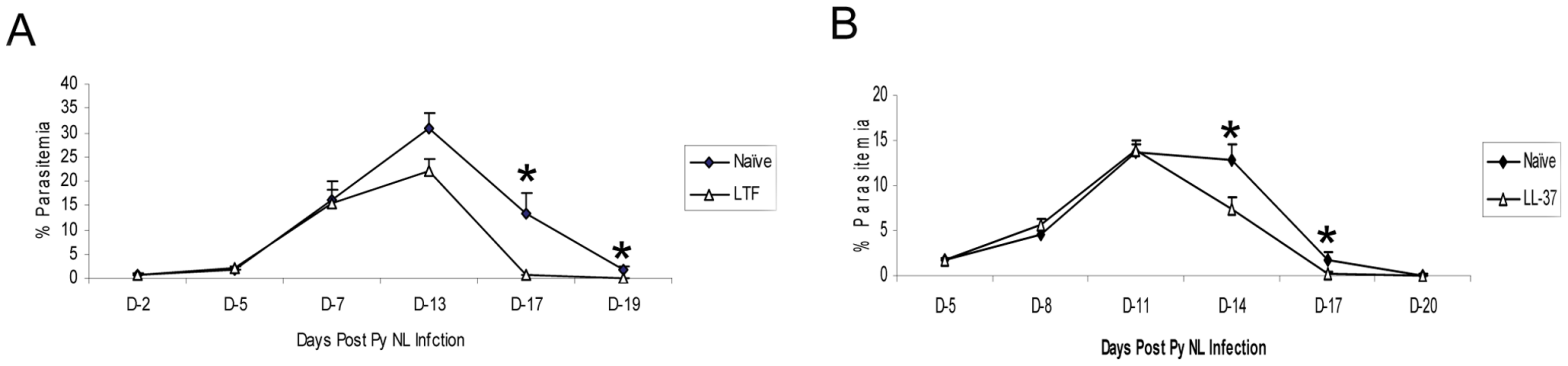
Anti-microbial peptide treatment decreases parasitemia levels in *P. yoelii* 17XNL infected mice. *P. yoelii* erythrocytic-stage parasites were incubated with lactoferrin (LTF - A) or the cathelicidin LL-37 (B) peptide for 1–2 hours prior to the malaria infection as described in the Methods section. One day later, animals in the treatment groups were given 50 micrograms of LTF or the LL-37 peptide. The post-infection parasitemia levels were determined by blood smear microscopy. These data are representative results from 2 experiments. Ten mice were used for each study group. * Significant difference in parasitemia levels (p<0.05) as determined by unpaired t test analysis.

The RT-PCR studies demonstrated that the *Ltf* and *Camp* genes were up-regulated in BCG vaccinated mice. To assess whether the exposure to antimicrobial peptides impacts the levels of post-infection malaria parasitemias in mice, erythrocytic *P. yoelii* 17XNL parasites were incubated for 1–2 hours with either lactoferrin or the cathelicidin LL-37 peptide prior to an IP infection. One day later, the infected mice were also directly injected with lactoferrin or the cathelicidin LL-37 peptide. (The LL-37 peptide is the human ortholog of the mouse CRAMP peptide. LL-37 and CRAMP are encoded by similar genes, have similar structures and exhibit similar antimicrobial activity and tissue distribution [21], [22], [23]). As shown in Figure 3A, reductions in the malaria infection were observed after the lactoferrin treatment. In these mice, significant 30% and 95% reductions (p<0.05) of malaria parasitemias were seen at days 14 and 17, respectively, relative to control *P. yoelii* 17XNL infected mice. Furthermore, modest but significant decreases in malaria parasitemias were detected after treatment with 50 µg/ml of the cathelicidin LL-37 peptide (Figure 3B). At day 14 following the erythrocytic malaria infection, a significant 43% reduction in *P.yoelii* parasitemias (p = 0.03) was seen in mice treated with the LL-37 peptide compared to infection controls.

## Discussion

Immunization strategies developed against childhood diseases generally aim to decrease the burden of infection by the pathogen targeted by the specific vaccine. Interestingly, considerable recent evidence suggests that vaccination may also provide non-specific benefits to the host; these non-specific effects may have significant public health consequences [24]. For example, the reduction in mortality observed in infants immunized with measles vaccine cannot be solely explained by decreased rates of measles infections [25]. Moreover, the overall increased survival seen in children with BCG scars in West Africa likely did not entirely result from improved control of tuberculosis [5]. While the non-specific impact of some vaccines has been demonstrated, the immune mechanisms of protection have generally not been elucidated. For BCG vaccine, it is assumed that the widespread consequences of immunization are related to BCG’s capacity to generate a Th1-type immune environment. However, the precise Th1 responses associated with the non-specific protection have not been defined.

In a seminal early study, Clark et al. showed that BCG immunization protected mice against lethal malaria infections (11). Although the mechanisms of the non-specific protections were not identified, the authors speculated that BCG-induced the release of non-antibody mediators of immunity from T lymphocytes. We wanted to further expand these initial observations and explore the molecular mechanisms that may contribute to the non-specific protection. Using a mouse malaria model, we investigated the non-specific immune responses induced by BCG vaccination. Initially, we demonstrated that immunization with BCG induced significant protection against a PyNL erythrocytic infection at 2 months post-infection. At days 14 and 16 after the PyNL infection, the levels of parasitemia were reduced 71% and 93%, respectively, in BCG vaccinated mice compared to naïve controls. Although we and others have shown that live BCG can be recovered from mice up to 2 years after standard subcutaneous injection of mice, it is unclear whether the presence of live organisms are required for the general anti-microbial effects of BCG [26, 27, S. Derrick, unpublished results]. Long-term drug therapy studies are planned to evaluate whether live BCG bacilli are needed for the non-specific protection. To determine whether T cells elicited the protective responses, we used cellular depletion strategies to investigate whether CD4 T cells and CD8 T cells are mediators of the BCG-induced protection. The CD4 depletion experiments were confounded by the critical role of CD4 T cells in limiting the progression of PyNL infections in naïve C57BL/6 mice. However, the loss of BCG-induced protection following CD8 T cell depletions show that CD8 T cells contribute to the anti-malarial activity evoked by BCG vaccination.

Importantly, the results of gene expression studies have suggested potential immunomolecules induced by BCG vaccination that may mediate non-specific antimicrobial immunity. As demonstrated in previous studies, BCG is a potent immunostimulant which can modulate the expression of numerous host genes [15], [16], [28]. In our experiments, BCG vaccination induced a broad array of immune molecules including 4 antimicrobial compounds, three IL-1 related molecules, and several chemokines. Of importance, the enhanced relative expression of the four genes encoding antimicrobial compounds (*Ltf* -lactoferrin, *Camp* - CRAMP cathelicidin, *Prg2*– major basic eosinophil protein, and *Epx*- eosinophil peroxidase) were maintained for at least 9 days after the immunosuppressive malaria infection. Each of these antimicrobial compounds plays a multifunctional role in host innate immunity and provides a first line of defense against invading microbes. The cathelicidin-type CRAMP peptide has been shown to have potent antibacterial activity against a variety of organisms including *M. tuberculosis*, *K. pneumoniae, S. aureus*, and *P. aeriginosa* [29], [30], [31], [32]. Furthermore, cathelicidin-type peptides can promote lymphocyte recruitment, dendritic mediated T cell activation, and the induction of pro-inflammatory cytokines and chemokines [19], [33]. Lactoferrin can also inhibit the growth of bacteria, viruses, and parasites by multiple mechanisms including sequestering iron, destabilizing microbial membranes, and interfering with microbial adherence to host cells [34]. Additionally, lactoferrin possesses a wide spectrum of immune modulating activities such as the promotion of T and B cell maturation, the activation of T cells, and the induction of Th1 cytokine responses. Directly relevant to our studies, lactoferrin has been shown to reduce invasion of *Plasmodium berghei* sporozoites into CHO cells by blocking binding of the malaria parasites and inhibit the *in vitro* growth of *P. falciparum* [35], [36]. Finally, release of the eosinophil granule proteins (eosinophil peroxidase and major basic protein) is clearly an important defense mechanism against filarial and malarial parasitic infections. Exposure to the major basic protein and eosinophil peroxidase has been shown to be toxic for many pathogens including *Schistosoma mansoni*, *Trichinella spiralis*, *Bruzea pahargi*, as well as *P. falciparum* [37], [38]. Overall, these antimicrobial molecules provide a critical link between innate and adaptive immunity. By inducing expression of these antimicrobial compounds and enhancing recruitment of eosinophils, BCG vaccination may provide an immune environment which could limit infections of many invading pathogens before effective host adaptive responses are generated [39], [40]. The initial experiment to validate this hypothesis in our mouse model involved preincubation of *P. yoelii* parasites with lactoferrin prior to the malaria challenge and then one day later parenteral injection of lactoferrin. Using these procedures, we showed that the lactoferrin treatment significantly reduced the levels of malaria parasitemia relative to naïve controls. Similar treatment protocolsshowed that a cathelicidin-type peptide also modestly but significantly decreased malaria parasitemia levels after the peak of the *P. yoelii* infection. Interestingly, both of these antimicrobial molecules did not have substantial effect on the early stages of the infection but did impact the later parasitemia levels. The absence of an early impact on the malaria infection suggests that these compounds did not directly inactivate the parasite during the pre-incubation period but likely activated cellular immune mechanisms which resulted in,a delayed anti-parasite response. Similar temporal antimicrobial cellular responses are seen in BCG-vaccinated mice infected with a low aerosol dose of *M. tuberculosis* [41]. In these experiments, differences in the pulmonary growth of the tuberculous infection between naïve and vaccinated mice are not detected until 7–10 days post-challenge. A pivotal role of cellular immune responses in the anti-parasitic activity of these antimicrobial peptides is consistent with the ablation of BCG-induced protection observed after CD8 T cell depletion. Further studies are ongoing to identify the precise cellular mechanisms such as Th1 cytokine induction or cytotoxic T cell activation, which are responsible for the antimicrobial peptide mediated protection.

In the mouse malaria model, BCG vaccination also reduced the suppression of gene expression by the malaria infection. It has been well established that malaria infections cause significant depressions of the host immune responses to concurrent infections and heterologous vaccination. Although the mechanisms underlying the immune hyporesponsiveness are uncertain, the induction of regulatory T cells, the inhibition of dendritic cell antigen presentation, and/or the rapid induction of IL-10 and TGF-β may contribute to immune suppression observed during malaria infections [42], [43], [44]. In this study, the expression of 11 genes was reduced in naive mice during the active malaria infection including the *Nos2*, *Camp*, *Ltf*, and *Pgr2* genes. In contrast, the relative expression of these immune mediators was not suppressed in malaria-infected BCG vaccinated mice compared to controls. The modulation of *Nos2* gene activity by BCG immunization is of particular interest because of the widely recognized role of inducible nitric oxide synthases in immune defense against intracellular pathogens [45]. Our preliminary studies in *Nos2* knockout mice (which show that the BCG anti-malarial activity is abrogated in the absence of *Nos2*) strongly support the involvement of nitric oxide in BCG-induced protection (M. Parra, data not shown).

Overall, we have shown that vaccination with BCG clearly induces expression of innate immune molecules including antimicrobial peptides which can provide protection against unrelated pathogens. Furthermore, we have demonstrated that the immune environment created by immunization with this potent immunostimulatory mycobacterial strain can diminish the suppressive immune effects seen after infection with malaria parasites. These data support the evidence which indicates that BCG immunization can provide overall health benefits to the vaccinated individual. Most importantly, the results of this study and related work emphasize that the removal of BCG from TB vaccination protocols may have a negative impact on public health and that public health officials should proceed with caution when contemplating the removal of BCG from new immunization protocols against TB. Furthermore, well designed clinical studies are warranted to study the impact of BCG immunization on malaria associated mortality and morbidity in low and high transmission areas.
